# Circulating S100B levels at birth and risk of six major neuropsychiatric or neurological disorders: a two-sample Mendelian Randomization Study

**DOI:** 10.1038/s41398-023-02478-3

**Published:** 2023-05-24

**Authors:** Mengyu Pan, James M. Roe, Ron Nudel, Andrew J. Schork, Olena Iakunchykova, Anders M. Fjell, Kristine B. Walhovd, Thomas Werge, Chi-hua Chen, Michael E. Benros, Yunpeng Wang

**Affiliations:** 1 Center for Lifespan Changes in Brain and Cognition (LCBC), Department of Psychology, University of Oslo, 0317 Oslo, Norway; 2 Copenhagen Research Centre for Mental Health, Mental Health Center Copenhagen, Copenhagen University Hospital, Gentofte Hospitalsvej 15, 2900 Hellerup, Denmark; 3 Neurogenomics Division, The Translational Genomics Research Institute (TGEN), 445 N. Fifth Street, 85004 Phoenix, AZ USA; 4 Institute of Biological Psychiatry, Mental Health Center St. Hans, Mental Health Services Copenhagen, Boserupvej 2, 4000 Roskilde, Denmark; 5 Division of Radiology and Nuclear Medicine, Oslo University Hospital, Rikshospitalet, POB 4950, Nydalen, 0424 Oslo, Norway; 6 Department of Clinical Medicine, University of Copenhagen, Blegdamsvej 9, 2100 Copenhagen, Denmark; 7 Department of Radiology, University of California in San Diego, Gilman Drive 9500, 92093 La Jolla, CA USA

**Keywords:** Depression, Predictive markers

## Abstract

Circulating levels of the astrocytic marker S100B have been associated with risk of neuropsychiatric or neurological disorders. However, reported effects have been inconsistent, and no causal relations have yet been established. We applied two-sample Mendelian Randomization (MR) on the association statistics from genome-wide association studies (GWAS) for circulating S100B levels measured 5-7 days after birth (the iPSYCH sample) and in an older adult sample (mean age, 72.5 years; the Lothian sample), upon those derived from major depression disorder (MDD), schizophrenia (SCZ), bipolar disorder (BIP), autism spectral disorder (ASD), Alzheimer’s disease (AD), and Parkinson’s disease (PD). We studied the causal relations in the two S100B datasets for risk of these six neuropsychiatric disorders. MR suggested increased S100B levels 5-7 days after birth to causally increase the risk of MDD (OR = 1.014; 95%CI = 1.007–1.022; FDR-corrected *p* = 6.43×10^−4^). In older adults, MR suggested increased S100B levels to have a causal relation to the risk of BIP (OR = 1.075; 95%CI = 1.026–1.127; FDR-corrected *p* = 1.35×10^−2^). No significant causal relations were found for the other five disorders. We did not observe any evidence for reverse causality of these neuropsychiatric or neurological disorders on altered S100B levels. Sensitivity analyses using more stringent SNP-selection criteria and three alternative MR models suggested the results are robust. Altogether, our findings imply a small cause-effect relation for the previously reported associations of S100B and mood disorders. Such findings may provide a novel avenue for the diagnosis and management of disorders.

## Background

Neuropsychiatric and neurological disorders are debilitating diseases with relatively high lifetime prevalence, incurring large burdens on families and society [1]. However, the causes of neuropsychiatric disorders are largely unknown. Recent large-scale GWAS have revealed a large number of genomic regions that associate with one or more of the mental disorders [2–7], but the exact causal genes underlying these disorders have proved hard to identify due to complex linkage disequilibrium (LD) in the genome. Nevertheless, aggregating association signals into biological pathways has uncovered several pathways that play key roles in the pathophysiology of mental disorders, such as synaptic regulations, neuroinflammation and glucose metabolism [2–7]. These pathways implicate not only neuronal but also glial cell dysfunction as a cause of neuropsychiatric disorders [8, 9].

The calcium-binding protein—S100B—is a classic marker for activated astrocytes. S100B levels in peripheral blood have been suggested as a biomarker for neuropsychiatric and neurological disorders [10, 11]. Although knowledge on the definitive biophysiological functions of S100B is incomplete, it has been demonstrated that S100B could contribute to intracellular and extracellular calcium homeostasis, cell survival and proliferation, and other enzymatic bioprocesses [12]. On the one hand, elevated circulating levels of S100B have been associated with risk of Schizophrenia (SCZ), bipolar disorders (BIP), major depression disorder (MDD), autism spectral disorders (ASD), and Alzheimer’s disease (AD) [11, 13–18]. However, others have also reported findings in the opposite direction [19, 20], or null associations [21]. As S100B levels have been shown to increase with age and be altered by the use of certain medications [22–24], differences in studied subjects may partly underlie conflicting results. Currently, it is not known whether alterations in blood S100B levels are causes or consequences of these disorders.

Mendelian randomization (MR) is a recently well-developed statistical framework for interrogating causal relations between an exposure and outcome [25, 26], based on observational data. Within this framework, the exposure variables of interest are indirectly randomized by ‘nature’ through genetic instrument variables (mostly single nucleotide polymorphisms (SNPs)) that strongly associate with the variation of exposures. Under certain assumptions, the average causal effects of exposures on outcomes can be estimated [27]. This framework is especially powerful if combined with the association statistics from large-scale GWAS using two-sample MR models [28]. The advantages of this include accurate effect size estimation using extremely large samples, and reduced confounder bias through measuring both exposure and outcomes in the same subjects.

Here, we present the first study investigating the causal effect of circulating S100B levels on the risk of six major neuropsychiatric or neurological disorders. We applied two-sample MR methods on the recently published large-scale GWAS for blood S100B levels measured 5-7 days after birth (N = 8138; the iPSYCH dataset) [29] and in an older adult sample (mean age: 72.5 years; *N* = 769; the Lothian dataset) [30, 31] as exposure, and case-control GWAS for SCZ (*N* = 79,845) [2], BIP(*N* = 51,710) [3], MDD(*N* = 500,199) [32], ASD(*N* = 46,350) [7], AD(*N* = 63,976), and Parkinson’s Disease (PD) (*N* = 482,730) [33] as outcomes. We further confirmed our findings by performing a range of sensitivity analysis varying the number of genetic instruments and using alternative MR models.

## Methods

### Study Aim and Design

The aim of the present study was to examine whether circulating levels of S100B are causally associated with the risk of neuropsychiatric disorders. To achieve this aim, we used an open-source two-sample MR program [28]. We took the association statistics of SNPs for our exposure – here S100B – and those for our outcomes SCZ, BIP, MDD, ASD, AD, and PD, as input. SNP association statistics were obtained from publicly accessible databases (see Availability of data and materials). This study follows the STROBE-MR guidelines [34].

### Exposure GWAS

GWAS for the circulating S100B levels were performed on dried blood spots samples collected 5-7 days after birth by the iPSYCH project [29, 35]. Levels of S100B were determined using the Meso-scale platform [36]. All participants to the GWAS are Danish, and 8,138 subjects in total were included in the GWAS. During the association analysis, age at 2012, sex and six genetic principal components (PCs) were included as covariates. The effects of SNPs were reported on the standard deviation of the log-transformed S100B levels in dried blood spots (1 SD = 3043 pg/mL). The second set of exposure statistics were derived from a GWAS of the Lothian Birth Cohort 1936, which included 769 participants (mean age 72.5 years). S100B levels from the blood for this sample were measured by the chemiluminescence immunoassay S100B kit (catalogue number 314701, distributed by DiaSorin, Berks, UK) [30]. The measured S100B levels were rank-based inverse transformed, and the effects of age, sex, and body mass index were regressed out before running GWAS [30].

### Outcome GWAS

GWAS results for the four psychiatric disorders were obtained with permission from the Psychiatric Genomics Consortium (PGC), each of which followed a case-control design and is an inverse-variance weighted meta-analysis across tens of participating GWAS sub-studies. All GWAS of sub-studies were performed using the Ricopili pipeline [37], thereby maintaining a consistent analysis protocol. For each sub-study GWAS, sex, age, and genetic principal components, the number of which varied between sub-studies, were included as covariates. Since our exposure GWAS were performed on mainly European populations, we only used GWAS results derived primarily from European ancestral samples, to reduce bias induced by ancestral differences. In addition, for the MDD GWAS, we used the dataset excluding samples from 23andMe.

The AD GWAS results were obtained through online application to the International Genomics of Alzheimer’s Project (IGAP) [5]. IGAP is a large three-stage study based upon genome-wide association studies (GWAS) on individuals of European ancestry. In stage 1, IGAP used genotyped and imputed data on 11,480,632 single nucleotide polymorphisms (SNPs) to meta-analyse GWAS datasets consisting of 21,982 Alzheimer’s disease cases and 41,944 cognitively normal controls from four consortia: The Alzheimer Disease Genetics Consortium (ADGC); The European Alzheimer’s disease Initiative (EADI); The Cohorts for Heart and Aging Research in Genomic Epidemiology Consortium (CHARGE); and The Genetic and Environmental Risk in AD Consortium Genetic and Environmental Risk in AD/Defining Genetic, Polygenic and Environmental Risk for Alzheimer’s Disease Consortium (GERAD/PERADES). In stage 2,11,632 SNPs were genotyped and tested for association in an independent set of 8362 Alzheimer’s disease cases and 10,483 controls. Meta-analysis of variants selected for analysis in stage 3 A (*n* = 11,666) or stage 3B (*n* = 30,511) samples brought the final sample to 35,274 clinical and autopsy-documented Alzheimer’s disease cases and 59,163 controls. In the present study, the stage 1 data was used.

The PD GWAS results were obtained from the fixed-effect meta-analysis performed by the International Parkinson Disease Genomics Consortium (IPDGC) [33]. This study included both patients and proxy-patients (i.e. those who were not diagnosed but their first-degree relatives were) as cases. A similar GWAS protocol was used by each sub-study before the meta-analysis. All individual sub-studies had adjusted for sex, age, and up to 10 PCs in their GWAS (see Nalls et al. [33]). In the present study, we used the association results excluding 23andMe samples.

### Instrumental SNPs selection

Prior to selecting instrumental SNPs, we quality checked the downloaded GWAS summary statistics. We removed variants with minor allele frequencies (MAF) < 0.05, and those that are not biallelic in the 1000 Genomes Project Phase 3 European samples(1KGP3), from all GWAS datasets. In addition, we removed SNPs that are ambiguous in allelic coding, i.e., A/T or C/G or were imputed with INFO score <0.6 when this information was available. To ensure comparability across disorders in terms of MR results, we removed SNPs that only exist in subsets of the GWAS results.

To select uncorrelated instrumental SNPs, we used PLINK [38] and the LD structure of the 1KGP3 dataset. The following parameters in PLINK were set, clump-kb 10,000 kb, -clump-p1 10^−6^, and -clump-r2 0.05. The association statistics for the instrumental SNPs to S100B and to each of the disorders were harmonized by assigning the same allele as the effective allele. The instrumental strengths of SNPs to S100B level was evaluated using the F-statistic [39]. SNPs having F-statistics >10 were considered as candidate for instruments. A full list of SNPs selected as instruments are presented in Supplementary Tables 1-30.

### Two-sample MR analysis

To test whether S100B levels have a causal effect on neuropsychiatric or neurological outcomes, we performed a two-sample MR analysis using S100B levels as exposure and each neuropsychiatric or neurological disorder as the outcome. To mitigate bias induced by horizontal pleiotropy (i.e., same genetic variants affecting both the exposure and outcome but through independent pathways) we first detected horizontal pleiotropic SNPs using the Mendelian Randomization Pleiotropy RESidual Sum and Outlier (MR-PRESSO) method [40]. Specifically, we used the MR-PRESSO global test to check whether there was horizontal pleiotropy in the instrumental SNP set; if the test was significant (*p* < 0.05), we used the MR-PRESSO outlier test to identify the pleiotropic SNPs and remove them from subsequent analysis. For our primary analysis we used the powerful invariance-weighted MR model (IVW) to detect causal effects. The Benjamini-Hochberg procedure was used to adjust multiple testing across disorders [41].

We then tested the possibility of reverse causality, namely that neuropsychiatric or neurological disorders have a causal effect on S100B levels, using each of the six disorders as exposure and S100B as the outcome in IVW-MR analysis.

### Sensitivity analysis

To confirm our results, we additionally applied three other two-sample MR methods: the MR-Egger regression, weighted median, and the MR robust adjusted profile score (MR-RAPS) [26]. To test if our results were due to the use of subgenome-wide significant SNPs (*p* < 10^−6^), we reran our analysis using SNPs that met the standard significance level, i.e., *p* < 5×10^−8^.

## Results

We detected horizontal pleiotropy between S100B levels and SCZ (*p* < 0.001), BIP (*p* < 0.01) and AD (*p* = 0.02), based on the MR-PRESSO global test. To mitigate bias induced by this, we removed SNPs flagged as pleiotropic (uncorrected *p* < 0.05) for SCZ (6 SNPs), BIP (2 SNPs), and AD (2 SNPS) using the MR-PRESSO outlier test. After removing these, we did not observe any evidence of horizontal pleiotropy (*p* > 0.05).

**Fig. 1:**
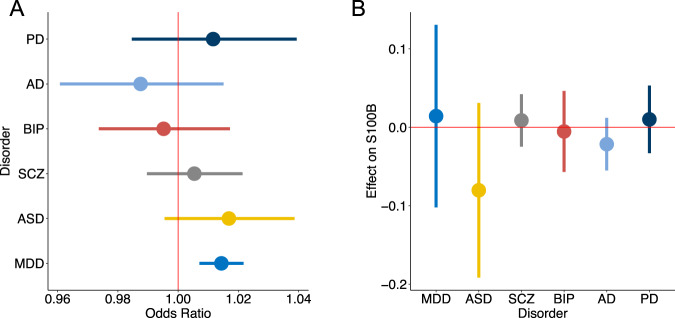
Bidirectional causal relations between S100B (iPSYCH) and six neuropsychiatric disorders. **A** Inverse variance weighted (IVW) MR models were used to estimate average causal effect of circulating levels of S100B measured in the iPSYCH sample on six neuropsychiatric disorders. Odds ratios and 95% confidence intervals are shown. **B** Causal effect of six neuropsychiatric disorders on S100B levels. Average effect size (beta) and 95% confidence intervals are shown. PD Parkinson’s disease, AD Alzheimer’s disease, BIP bipolar disorder, SCZ Schizophrenia, ASD autism spectral disorders, MDD major depression disorder.

We then applied IVW-MR on each of the six neuropsychiatric or neurological disorders. Figure 1 shows that increased S100B levels 5–7 days after birth were causally associated with increased risk of MDD (Odds ratio (OR) = 1.014; 95%CI = 1.007–1.022; FDR-corrected *p* = 6.43×10^−4^). We detected no significant causal effects of S100B on the risk to SCZ, BIP, ASD, AD and PD (*p* > 0.05, Table 1). The significant causal effect of S100B on MDD was also detected by the MR-RAPs and weighted median methods, both of which showed very similar effect sizes (Supplementary Table 31), whereas the Egger-regression method showed only a trend (OR = 1.012, 95%CI = 0.9988–1.027; uncorrected *p* = 0.0835). Using more significant instrumental SNPs (association *p* < 5×10^−8^), we again found significant causal effects of S100B on MDD by IVW, MR-RAPs and weighted median methods (Supplementary Table 32). For the other five neuropsychiatric or neurological disorders, we again detected no significant causal relations for any of these three methods, also at the stringent association threshold.

**Table 1:** Causal effects of S100B levels measure at birth on neuropsychiatric disorders.

Outcome	NSNP	OR	OR_95%CI	Het_p	MR-PRESSO	pval	padj
SCZ	34	1.005	0.990–1.021	0.580	0.613	0.504	1.0
BIP	32	0.995	0.974–1.017	0.454	0.347	0.666	1.0
ASD	37	1.017	0.996–1.039	0.294	0.362	0.122	0.612
MDD	33	1.014	1.007–1.022	0.162	0.248	1.07 × 10^−4^	**6.43** **×** **10**^**−4**^
AD	44	0.988	0.961–1.015	0.109	0.121	0.372	1.0
PD	40	1.012	0.985–1.039	0.433	0.448	0.402	1.0

*SCZ* Schizophrenia, *BIP* bipolar disorder, *ASD* autism spectral disorders, *MDD* major depression disorder, *AD* Alzheimer’s disease, *PD* Parkinson’s disease, *NSNP* number of instrumental SNPs selected, *OR* odds ratio for disorders, *OR_95%CI* the 95% confidence interval for OR, *Het_p* *p* value of the test of heterogeneity of instrumental SNP, *MR_PRESSO* *p* value of the global horizontal pleiotropy test, *pval* *p* value for the MR analysis, *padj* Benjamini-Hochberg False discovery rate adjusted *p* value.

The causal effects of S100B level measure in the iPSYCH sample on six neuropsychiatric disorders were estimated by the Inverse Variance Weighted MR method. Instrumental SNPs for S100B were selected from SNPs that associates with S100B levels at *p* < 10^−6^.

In the reversed direction, we did not find any causal effects of neuropsychiatric or neurological disorders on S100B levels 5-7 days after birth (Fig. 1B). Neither did the other three MR methods find any relations (Supplementary Table 33). Using the stringent association threshold for instrumental SNPs (*p* < 5 × 10^−8^) still gave the same negative results (Supplementary Table 34).

**Fig. 2:**
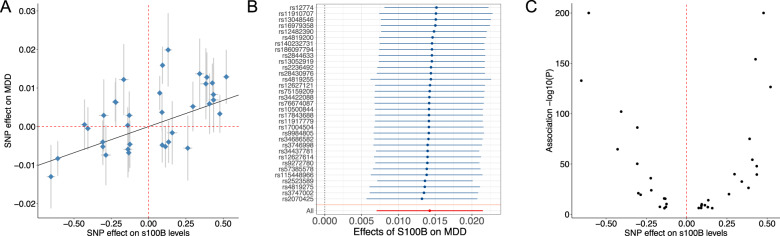
Effects of instrumental SNPs on MDD and S100B (iPSYCH). **A** Effects of selected instrumental SNPs on MDD (on the scale of log (odds ratio)) and on S100B levels (on the scale of standard deviation of S100B levels) measured in the iPSYCH sample. Confidence interval (95%) are also shown. **B** IVW estimates for the effect of S100B on MDD after excluding each of the SNPs on the y axis, i.e., leave-one-out. The overall effect is indicated at the bottom of the panel (All). **C** Volcano plot for the effect of instrument SNPs on S100B levels shows a symmetric pattern about the zero line.

We then investigated the details of the causal effect of S100B on MDD risk (see Fig. 2). We found that a one-standard deviation (SD) increase in S100B levels measured 5-7 days after birth increases the risk of MDD later in life by an odds ratio of 1.014 (95%CI = 1.007–1.022; Fig. 2A). The instrumental SNPs used to detect this relation were all consistent in direction of effect (as shown in the leave-one-out analysis (Fig. 2B)), did not show significant heterogeneity in the effect of S100B on MDD (Cohran’s heterogeneity, *p* = 0.162), and their effect on S100B levels were close to symmetric around the zero line (Fig. 2C). These diagnostic results imply the causal effect of S100B on MDD is reliable.

**Fig. 3:**
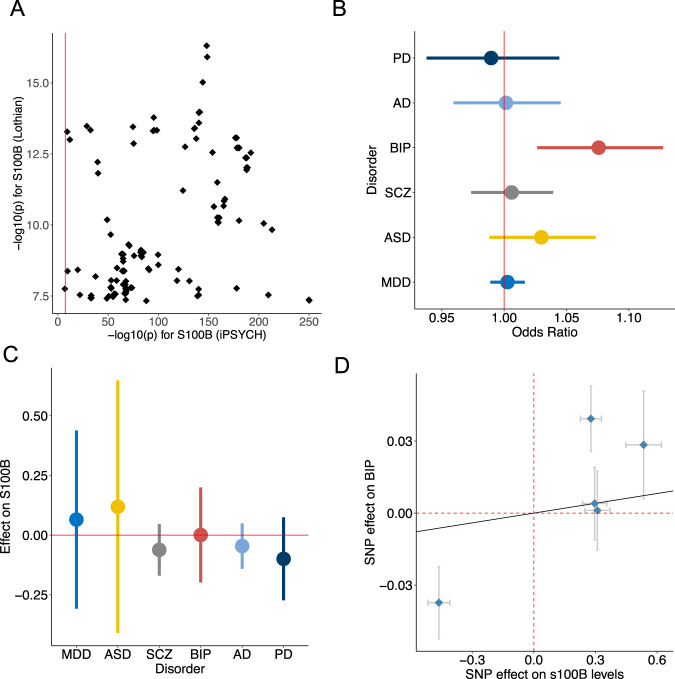
Bidirectional causal relations between S100B (Lothian) and six neuropsychiatric disorders. **A** Comparison of association strengths for SNPs in the Lothian and iPSYCH sample. SNPs that have been discovered in the Lothian study (*p* < 5e-8) are shown. **B** IVW MR models were used to estimate average causal effect of circulating levels of S100B measured in the Lothian sample on six neuropsychiatric disorders. Odds ratios and 95% confidence intervals are shown. **C** Causal effect of six neuropsychiatric disorders on S100B levels. Average effect size (beta) and 95% confidence intervals are shown. **D** Effects of instrumental SNPs on S100B levels and risk of BIP. PD Parkinson’s disease, AD Alzheimer’s disease, BIP bipolar disorder, SCZ Schizophrenia, ASD autism spectral disorders, MDD major depression disorder.

We next compared the association results for S100B levels measured 5-7 days after birth in dried blood spots (the iPSYCH data) to that measured in an older adults sample (the Lothian data) [30]. In total, 156 SNPs existing in both studies were significant in the Lothian data (*p* < 5 × 10^−8^), all of which were also significantly associated with S100B in the iPSYCH data (Fig. 3A). However, the association strengths were much stronger in the iPSYCH data than in the Lothian data. Moreover, more than half of these SNPs showed the opposite direction of effects between the two studies (Supplementary Table 35). Altogether, this implies while the same genetic variants influence the circulating levels of S100B, their effect directions and strengths vary with age.

We then applied our protocol of two-sample MR analysis using the Lothian S100B GWAS results as exposure. Due to the small sample size of this GWAS, very few SNPs passed our selection criteria as instrumental SNPs for S100B upon each disorder (<5, Supplementary Tables 25-30). The causal effect of S100B on MDD, discovered above, was not significant in this data. However, we did find a significant causal effect of S100B on the other mood disorder, BIP: a one SD increase in S100B levels increased the odds ratio of BIP by 1.07 (95%CI: 1.03–1.13; *p* = 0.002; Fig. 3D). This causal relation was also supported by the weighted median and MR-RAPs methods (Supplementary Table 36). In line with the results from the iPSYCH data, no causal relations were found for the other five disorders, nor for the reverse direction of causality in any disorder (Fig. 3C, Supplementary Tables 37 and 38).

## Discussion

We present a comprehensive study aimed at identifying the causal effects of circulating S100B levels on six neuropsychiatric or neurological disorders, including neurodevelopmental (ASD and SCZ), neurodegenerative (AD and PD), and mood disorders (BIP and MDD). We used two datasets for S100B, one measured 5-7 days after birth and the other for those aged over 70 years. We show that increased S100B levels after birth may causally raise the lifetime risk of MDD, a relation that did not exist for S100B levels measured after 70 years of age. However, we did find that increased S100B levels after 70 years of age had a causal effect on the risk of BIP—the other mood disorder included in our study. Thus, both datasets pointed to a causal effect of increased blood S100B levels on the lifetime risk of mood disorders, but not on neurodevelopmental or neurodegenerative disorders. Therefore, future mechanistic studies on the effect of S100B on this group of mental disorders are warranted.

Several reasonings may explain the different findings from the two S100B datasets. Foremost, S100B levels measured 5–7 days after birth may be under the stronger genetic influence than that measured later in life, as reflected by an extremely high SNP-based heritability (h^2^ = 0.7) in our recent iPSYCH-based GWAS results [29]. Therefore, using SNP instruments will suffer less from unknown environmental confounding on the causal relations between S100B and neuropsychiatric or neurological disorders. Second, it is known that blood levels of S100B could be altered by injury to the brain, medication, and other conditions [22, 23, 42]. Under these conditions, the effect of genetic variants on S100B levels may be different than right after birth. This hypothesis is supported by the comparison of the two S100B GWAS results, which showed similar associations but varying effects, with the caution of incomparable sample sizes for the two. Last, the small sample for the Lothian GWAS may be underpowered to identify enough instrumental SNPs for S100B levels, as less than five SNPs could be selected given our criteria. Thus, when large samples become available, the effect of S100B on neuropsychiatric or neurological disorders will become clearer for this age group.

S100B is a small protein (10–12 kDa), can pass through the blood-brain barrier, and is primarily produced by astrocytes [43]. While it can also be secreted to a lesser extent by adipocytes and possibly other cells, its decay rate from these sources is higher than from astrocytes [43, 44]. Therefore, blood circulating levels of S100B are most likely to be of astrocyte origin. In the brain, at low intracellular concentrations (e.g., nanomolar level), S100B showed neurotrophic effects in vitro, promoting neuronal differentiation, growth and survival [45–47]. But at increased extracellular concentrations it showed neurotoxic effects. While we still lack a mechanistic understanding for this biological phenomenon, it has been proposed that S100B may bind to receptor for advanced glycation end products (RAGE) and induce apoptosis processes in neurons, astrocytes and other glial cells, thereby increasing levels of harmful reactive species in the local extracellular environment. High extracellular levels for S100B can also activate surrounding microglia and astrocytes to promote an inflammatory cellular stage [44]. Although all these biological processes have been implicated as causal pathways leading to neuropsychiatric or neurological disorders, more confirmative data in vivo is still needed.

Surprisingly, we did not observe any causal effect of S100B on four of the neuropsychiatric or neurological disorders studied. On the one hand, our negative result may suggest previously reported associations were influenced by some unmeasured confounding variables. On the other hand, the true causal effect of S100B on these disorders seems very small, such that larger datasets are needed to delineate the effects. We also cannot rule out the third possibility that S100B may causally affect the risk of neuropsychiatric or neurological disorders during some critical windows in the life course outside of the neonatal and older age-ranges measured here, or that the elevated levels of S100B as measured in several studies of neuropsychiatric or neurological disorders are due to other underlying causes than S100B gene variation.

### Strengths and limitations

We used the largest GWAS results to date for circulating S100B levels. For the six neuropsychiatric or neurological disorders, we made a trade-off between sample size and the portion of clinical samples included in the original GWAS. We emphasized the latter for ease of interpretation. In addition, the use of S100B measured 5–7 days after birth facilitated a natural cause-effect interpretation in the temporal aspect for disorders developed later in life, which further strengthens our two-sample MR results. However, in this birth sample, S100B was measured in dried blood spots such that its exact levels were not comparable to those measured in serum or whole blood, the material used in the Lothian sample. For this reason, we interpreted the effect of S100B on the unit of standard deviation (SD = 3043 pg/mL) [36]. Because the GWAS of S100B in the Lothian sample were rank-based normalized before analysis [30], our estimated causal effect could not be transferred to other samples. Therefore, our results should be interpreted as evidence of the existence of such a causal effect, but the effect sizes may not be generalizable.

## Conclusion

Our data indicates increased circulating S100B levels may causally associate with the lifetime risk of mood disorders. Hence, our findings point to a potential causal explanation behind previously reported S100B associations with brain disorders, possibly opening avenues for novel treatments of major depression and bipolar disorders.

## Supplementary information

Supplementary Table 1-6

Supplementary Table 7-12

Supplementary Table 13-18

Supplementary Table 19-24

Supplementary Table 25-30

Supplementary Table 31-34

## Data Availability

The datasets supporting the conclusions of this article are publicly available at S100B GWAS results: http://www.ipsych.dk Psychiatric disorder GWAS results: https://www.med.unc.edu/pgc/download-results Alzheimer’s disease GWAS results: https://www.niagads.org/igap-rv-summary-stats-kunkle-p-value-data Parkinson’s disease GWAS results: https://drive.google.com/drive/folders/10bGj6HfAXgl-JslpI9ZJIL_JIgZyktxn Mendelian randomization software Two-Sample MR: https://mrcieu.github.io/TwoSampleMR/ MR-RAPS: https://github.com/qingyuanzhao/mr.raps/tree/multivariate MR-PRESSO: https://github.com/rondolab/MR-PRESSO.
