# Supplementary material for: Circulating S100B levels at birth and risk of six major neuropsychiatric or neurological disorders: a two-sample Mendelian Randomization Study
Source: Transl Psychiatry. 2023 May 24;13:174. doi: 10.1038/s41398-023-02478-3 (PMC10209162; doi:10.1038/s41398-023-02478-3)
Supplement: Supplementary file 6 — Supplementary Table 31-34 [file 41398_2023_2478_MOESM6_ESM.docx]

**Supplementary Information**

Circulating S100B Levels at Birth and Risk of six Major Neuropsychiatry Disorders: A Two-sample Mendelian Randomization Study

Pan et al

Table of Contents

[Table S31. Two-Sample MR: S100B to disorders, instrumental SNPs with p<1x10^-6^ 2](#_Toc93581422)

[Table S32. Two-Sample MR: S100B to disorders, instrumental SNPs with p<5x10^-8^ 3](#_Toc93581423)

[Table S33. Two-Sample MR: disorders to S100B, instrumental SNPs with p<1x10^-6^ 4](#_Toc93581424)

[Table S34. Two-Sample MR: disorders to S100B, instrumental SNPs with p<5x10^-8^ 5](#_Toc93581425)

[Table S36. Two-Sample MR: Lothian cohort S100B to disorders, instrumental SNPs with p<1x10^-6^ 6](#_Toc93581426)

[Table S37. Two-Sample MR: disorders to Lothian S100B, instrumental SNPs with p<1x10^-6^ 7](#_Toc93581427)

[Table S38. Two-Sample MR: disorders to Lothian S100B, instrumental SNPs with p<1x10^-8^ 8](#_Toc93581428)

# Table S31. Two-Sample MR: S100B to disorders, instrumental SNPs with p<1x10^-6^

| Outcome | Method | NSNP | OR | OR_CI_L | OR_CI_R | Pval |
| --- | --- | --- | --- | --- | --- | --- |
| SCZ | MR Egger | 34 | 0.9904 | 0.9623 | 1.0193 | 0.5156 |
| SCZ | Inverse variance weighted | 34 | 1.0054 | 0.9896 | 1.0214 | 0.5045 |
| SCZ | Weighted median | 34 | 0.9975 | 0.9759 | 1.0194 | 0.8193 |
| SCZ | Robust adjusted profile score (RAPS) | 34 | 1.0046 | 0.9883 | 1.0211 | 0.5843 |
| BIP | MR Egger | 32 | 1.0190 | 0.9808 | 1.0586 | 0.3423 |
| BIP | Inverse variance weighted | 32 | 0.9952 | 0.9737 | 1.0172 | 0.6660 |
| BIP | Weighted median | 32 | 1.0005 | 0.9698 | 1.0322 | 0.9755 |
| BIP | Robust adjusted profile score (RAPS) | 32 | 0.9941 | 0.9720 | 1.0166 | 0.6038 |
| ASD | MR Egger | 37 | 0.9844 | 0.9513 | 1.0187 | 0.3748 |
| ASD | Inverse variance weighted | 37 | 1.0169 | 0.9955 | 1.0387 | 0.1223 |
| ASD | Weighted median | 37 | 1.0124 | 0.9822 | 1.0435 | 0.4243 |
| ASD | Robust adjusted profile score (RAPS) | 37 | 1.0149 | 0.9917 | 1.0386 | 0.2093 |
| MDD | MR Egger | 33 | 1.0126 | 0.9988 | 1.0267 | 0.0835 |
| MDD | Inverse variance weighted | 33 | 1.0144 | 1.0071 | 1.0217 | 0.0001 |
| MDD | Weighted median | 33 | 1.0143 | 1.0049 | 1.0238 | 0.0028 |
| MDD | Robust adjusted profile score (RAPS) | 33 | 1.0144 | 1.0067 | 1.0222 | 0.0002 |
| AD | MR Egger | 44 | 1.0452 | 1.0016 | 1.0907 | 0.0483 |
| AD | Inverse variance weighted | 44 | 0.9875 | 0.9607 | 1.0151 | 0.3721 |
| AD | Weighted median | 44 | 1.0053 | 0.9759 | 1.0357 | 0.7256 |
| AD | Robust adjusted profile score (RAPS) | 44 | 0.9866 | 0.9587 | 1.0153 | 0.3562 |
| PD | MR Egger | 40 | 1.0249 | 0.9779 | 1.0742 | 0.3103 |
| PD | Inverse variance weighted | 40 | 1.0116 | 0.9846 | 1.0394 | 0.4018 |
| PD | Weighted median | 40 | 1.0125 | 0.9751 | 1.0513 | 0.5190 |
| PD | Robust adjusted profile score (RAPS) | 40 | 1.0097 | 0.9822 | 1.0379 | 0.4937 |

NSNP, number of instrumental SNPs selected; OR, odds ratio for disorders; OR_CI_L, left bound for 95% confidence interval for OR; OR_CI_R, right bound for 95% confidence interval for OR. Significant results for MDD are in shade.

# Table S32. Two-Sample MR: S100B to disorders, instrumental SNPs with p<5x10^-8^

| Outcome | Method | NSNP | OR | OR_CI_L | OR_CI_R | Pval |
| --- | --- | --- | --- | --- | --- | --- |
| SCZ | MR Egger | 36 | 0.9822 | 0.9352 | 1.0317 | 0.4800 |
| SCZ | Inverse variance weighted | 36 | 1.0076 | 0.9811 | 1.0349 | 0.5763 |
| SCZ | Weighted median | 36 | 0.9981 | 0.9763 | 1.0205 | 0.8691 |
| SCZ | Robust adjusted profile score (RAPS) | 36 | 1.0085 | 0.9826 | 1.0350 | 0.5243 |
| BIP | MR Egger | 33 | 1.0010 | 0.9435 | 1.0621 | 0.9729 |
| BIP | Inverse variance weighted | 33 | 1.0017 | 0.9723 | 1.0320 | 0.9086 |
| BIP | Weighted median | 33 | 1.0012 | 0.9695 | 1.0340 | 0.9403 |
| BIP | Robust adjusted profile score (RAPS) | 33 | 1.0029 | 0.9729 | 1.0339 | 0.8503 |
| ASD | MR Egger | 31 | 0.9845 | 0.9479 | 1.0226 | 0.4264 |
| ASD | Inverse variance weighted | 31 | 1.0148 | 0.9938 | 1.0362 | 0.1681 |
| ASD | Weighted median | 31 | 1.0123 | 0.9832 | 1.0423 | 0.4119 |
| ASD | Robust adjusted profile score (RAPS) | 31 | 1.0132 | 0.9899 | 1.0372 | 0.2673 |
| MDD | MR Egger | 29 | 1.0190 | 1.0044 | 1.0338 | 0.0164 |
| MDD | Inverse variance weighted | 29 | 1.0135 | 1.0064 | 1.0207 | 0.0002 |
| MDD | Weighted median | 29 | 1.0143 | 1.0046 | 1.0240 | 0.0037 |
| MDD | Robust adjusted profile score (RAPS) | 29 | 1.0136 | 1.0061 | 1.0212 | 0.0004 |
| AD | MR Egger | 36 | 1.0509 | 1.0094 | 1.0942 | 0.0213 |
| AD | Inverse variance weighted | 36 | 0.9909 | 0.9670 | 1.0154 | 0.4620 |
| AD | Weighted median | 36 | 1.0055 | 0.9765 | 1.0354 | 0.7140 |
| AD | Robust adjusted profile score (RAPS) | 36 | 0.9906 | 0.9651 | 1.0167 | 0.4761 |
| PD | MR Egger | 33 | 1.0204 | 0.9642 | 1.0799 | 0.4905 |
| PD | Inverse variance weighted | 33 | 1.0135 | 0.9848 | 1.0430 | 0.3598 |
| PD | Weighted median | 33 | 1.0125 | 0.9753 | 1.0512 | 0.5152 |
| PD | Robust adjusted profile score (RAPS) | 33 | 1.0129 | 0.9827 | 1.0439 | 0.4071 |

NSNP, number of instrumental SNPs selected; OR, odds ratio for disorders; OR_CI_L, left bound for 95% confidence interval for OR; OR_CI_R, right bound for 95% confidence interval for OR. Significant results for MDD are in shade.

# Table S33. Two-Sample MR: disorders to S100B, instrumental SNPs with p<1x10^-6^

| Exposure | method | NSNP | beta | se | Pval |
| --- | --- | --- | --- | --- | --- |
| SCZ | MR Egger | 213 | 0.0903 | 0.0712 | 0.2059 |
| SCZ | Inverse variance weighted | 213 | 0.0088 | 0.0171 | 0.6077 |
| SCZ | Weighted median | 213 | 0.0303 | 0.0228 | 0.1848 |
| SCZ | Robust adjusted profile score (RAPS) | 213 | 0.0181 | 0.0181 | 0.3162 |
| BIP | MR Egger | 59 | -0.0861 | 0.1456 | 0.5566 |
| BIP | Inverse variance weighted | 59 | -0.0053 | 0.0263 | 0.8398 |
| BIP | Weighted median | 59 | 0.0198 | 0.0383 | 0.6045 |
| BIP | Robust adjusted profile score (RAPS) | 59 | -0.0093 | 0.0279 | 0.7392 |
| ASD | MR Egger | 13 | -0.2009 | 0.3075 | 0.5271 |
| ASD | Inverse variance weighted | 13 | -0.0803 | 0.0568 | 0.1575 |
| ASD | Weighted median | 13 | -0.1173 | 0.0742 | 0.1138 |
| ASD | Robust adjusted profile score (RAPS) | 13 | -0.0809 | 0.0590 | 0.1703 |
| MDD | MR Egger | 133 | 0.2402 | 0.2962 | 0.4189 |
| MDD | Inverse variance weighted | 133 | 0.0142 | 0.0594 | 0.8105 |
| MDD | Weighted median | 133 | 0.0111 | 0.0769 | 0.8849 |
| MDD | Robust adjusted profile score (RAPS) | 133 | -0.0109 | 0.0604 | 0.8566 |
| AD | MR Egger | 48 | 0.0222 | 0.0320 | 0.4913 |
| AD | Inverse variance weighted | 48 | -0.0215 | 0.0171 | 0.2081 |
| AD | Weighted median | 48 | -0.0054 | 0.0224 | 0.8091 |
| AD | Robust adjusted profile score (RAPS) | 48 | -0.0162 | 0.0167 | 0.3308 |
| PD | MR Egger | 30 | 0.05516 | 0.0586 | 0.3545 |
| PD | Inverse variance weighted | 30 | 0.0101 | 0.0220 | 0.6466 |
| PD | Weighted median | 30 | 0.0211 | 0.0315 | 0.5022 |
| PD | Robust adjusted profile score (RAPS) | 30 | 0.0074 | 0.0230 | 0.7483 |

NSNP, number of instrumental SNPs selected; beta, effect of disorders on S100B; se, standard error for beta; Pval, p value for MR causal effects.

# Table S34. Two-Sample MR: disorders to S100B, instrumental SNPs with p<5x10^-8^

| Exposure | Method | NSNP | beta | se | pval |
| --- | --- | --- | --- | --- | --- |
| SCZ | MR Egger | 111 | 0.1410 | 0.0844 | 0.0979 |
| SCZ | Inverse variance weighted | 111 | 0.0076 | 0.0202 | 0.7076 |
| SCZ | Weighted median | 111 | 0.0245 | 0.0298 | 0.4113 |
| SCZ | Robust adjusted profile score (RAPS) | 111 | 0.0086 | 0.0220 | 0.6971 |
| BIP | MR Egger | 11 | -0.0617 | 0.3820 | 0.8752 |
| BIP | Inverse variance weighted | 11 | -0.0066 | 0.0560 | 0.9061 |
| BIP | Weighted median | 11 | 0.0471 | 0.0746 | 0.5278 |
| BIP | Robust adjusted profile score (RAPS) | 11 | 0.0038 | 0.0583 | 0.9477 |
| MDD | MR Egger | 54 | 0.8409 | 0.3913 | 0.0363 |
| MDD | Inverse variance weighted | 54 | 0.1166 | 0.0754 | 0.1224 |
| MDD | Weighted median | 54 | 0.1077 | 0.1083 | 0.3197 |
| MDD | Robust adjusted profile score (RAPS) | 54 | 0.1146 | 0.0786 | 0.1451 |
| AD | MR Egger | 32 | 0.0246 | 0.0349 | 0.4873 |
| AD | Inverse variance weighted | 32 | -0.0187 | 0.0176 | 0.2866 |
| AD | Weighted median | 32 | -0.0067 | 0.0220 | 0.7611 |
| AD | Robust adjusted profile score (RAPS) | 32 | -0.0146 | 0.0174 | 0.3995 |
| PD | MR Egger | 20 | 0.0565 | 0.0656 | 0.4002 |
| PD | Inverse variance weighted | 20 | 0.0223 | 0.0243 | 0.3592 |
| PD | Weighted median | 20 | 0.0232 | 0.0343 | 0.4989 |
| PD | Robust adjusted profile score (RAPS) | 20 | 0.0178 | 0.0252 | 0.4796 |

NSNP, number of instrumental SNPs selected; beta, effect of disorders on S100B; se, standard error for beta; Pval, p value for MR causal effects. Since only two SNPs met our criteria as instrumental SNPs for ASD, we did not perform analysis for this disorder.

# Table S36. Two-Sample MR: Lothian cohort S100B to disorders, instrumental SNPs with p<1x10^-6^

| Outcome | Method | NSNP | OR | OR_CI_L | OR_CI_R | Pval |
| --- | --- | --- | --- | --- | --- | --- |
| SCZ | MR Egger | 4 | 1.1146 | 0.9808 | 1.2666 | 0.2382 |
| SCZ | Inverse variance weighted | 4 | 1.0059 | 0.9736 | 1.0394 | 0.7226 |
| SCZ | Weighted median | 4 | 1.0084 | 0.9693 | 1.0492 | 0.6782 |
| SCZ | Robust adjusted profile score (RAPS) | 4 | 1.0060 | 0.9721 | 1.0411 | 0.7305 |
| BIP | MR Egger | 4 | 1.0289 | 0.8304 | 1.2748 | 0.8191 |
| BIP | Inverse variance weighted | 4 | 1.0758 | 1.0265 | 1.1275 | 0.0023 |
| BIP | Weighted median | 4 | 1.0721 | 1.0176 | 1.1296 | 0.0089 |
| BIP | Robust adjusted profile score (RAPS) | 4 | 1.0757 | 1.0243 | 1.1297 | 0.0034 |
| ASD | MR Egger | 4 | 1.0452 | 0.8890 | 1.2289 | 0.6457 |
| ASD | Inverse variance weighted | 4 | 1.0298 | 0.9881 | 1.0733 | 0.1636 |
| ASD | Weighted median | 4 | 1.0433 | 0.9912 | 1.0981 | 0.1046 |
| ASD | Robust adjusted profile score (RAPS) | 4 | 1.0301 | 0.9866 | 1.0755 | 0.1776 |
| MDD | MR Egger | 5 | 1.0192 | 0.9589 | 1.0832 | 0.5845 |
| MDD | Inverse variance weighted | 5 | 1.0027 | 0.9889 | 1.0166 | 0.7048 |
| MDD | Weighted median | 5 | 0.9964 | 0.9810 | 1.0121 | 0.6514 |
| MDD | Robust adjusted profile score (RAPS) | 5 | 1.0016 | 0.9887 | 1.0147 | 0.8085 |
| AD | MR Egger | 4 | 1.0625 | 0.8990 | 1.2556 | 0.5509 |
| AD | Inverse variance weighted | 4 | 1.0015 | 0.9594 | 1.0454 | 0.9468 |
| AD | Weighted median | 4 | 1.0084 | 0.9561 | 1.0634 | 0.7593 |
| AD | Robust adjusted profile score (RAPS) | 4 | 1.0015 | 0.9581 | 1.0468 | 0.9482 |
| PD | MR Egger | 5 | 0.8677 | 0.7011 | 1.0739 | 0.2832 |
| PD | Inverse variance weighted | 5 | 0.9896 | 0.9378 | 1.0442 | 0.7024 |
| PD | Weighted median | 5 | 0.9786 | 0.9174 | 1.0440 | 0.5125 |
| PD | Robust adjusted profile score (RAPS) | 5 | 0.9895 | 0.9356 | 1.0464 | 0.7105 |

NSNP, number of instrumental SNPs selected; OR, odds ratio for disorders; OR_CI_L, left bound for 95% confidence interval for OR; OR_CI_R, right bound for 95% confidence interval for OR.

# Table S37. Two-Sample MR: disorders to Lothian S100B, instrumental SNPs with p<1x10^-6^

| Exposure | Method | NSNP | beta | se | pval |
| --- | --- | --- | --- | --- | --- |
| SCZ | MR Egger | 201 | 0.2225 | 0.2310 | 0.3366 |
| SCZ | Inverse variance weighted | 201 | -0.0619 | 0.0553 | 0.2626 |
| SCZ | Weighted median | 201 | -0.0111 | 0.0790 | 0.8885 |
| SCZ | Robust adjusted profile score (RAPS) | 201 | -0.0568 | 0.0582 | 0.32890 |
| BD | MR Egger | 60 | 0.2072 | 0.5514 | 0.7084 |
| BD | Inverse variance weighted | 60 | 0.0006 | 0.1018 | 0.9956 |
| BD | Weighted median | 60 | 0.0020 | 0.1315 | 0.9880 |
| BD | Robust adjusted profile score (RAPS) | 60 | -0.0505 | 0.1049 | 0.6304 |
| ASD | MR Egger | 11 | -1.7690 | 1.2823 | 0.2010 |
| ASD | Inverse variance weighted | 11 | 0.1187 | 0.2701 | 0.6602 |
| ASD | Weighted median | 11 | 0.1173 | 0.3097 | 0.7048 |
| ASD | Robust adjusted profile score (RAPS) | 11 | 0.1703 | 0.2614 | 0.5148 |
| MDD | MR Egger | 131 | -2.2706 | 0.9313 | 0.0161 |
| MDD | Inverse variance weighted | 131 | 0.0650 | 0.1905 | 0.7329 |
| MDD | Weighted median | 131 | 0.0821 | 0.2709 | 0.7619 |
| MDD | Robust adjusted profile score (RAPS) | 131 | 0.1112 | 0.2034 | 0.5846 |
| AD | MR Egger | 47 | 0.1222 | 0.0920 | 0.1910 |
| AD | Inverse variance weighted | 47 | -0.0459 | 0.0487 | 0.3456 |
| AD | Weighted median | 47 | -0.0070 | 0.0787 | 0.9296 |
| AD | Robust adjusted profile score (RAPS) | 47 | -0.0560 | 0.0504 | 0.2662 |
| PD | MR Egger | 30 | -0.0774 | 0.2433 | 0.7527 |
| PD | Inverse variance weighted | 30 | -0.0995 | 0.0887 | 0.2619 |
| PD | Weighted median | 30 | -0.0844 | 0.1164 | 0.4684 |
| PD | Robust adjusted profile score (RAPS) | 30 | -0.0910 | 0.0921 | 0.3235 |

NSNP, number of instrumental SNPs selected; beta, effect of disorders on S100B; se, standard error for beta; Pval, p value for MR causal effects.

# Table S38. Two-Sample MR: disorders to Lothian S100B, instrumental SNPs with p<1x10^-8^

| Exposure | Method | NSNP | beta | se | pval |
| --- | --- | --- | --- | --- | --- |
| SCZ | MR Egger | 104 | 0.1514 | 0.2869 | 0.5988 |
| SCZ | Inverse variance weighted | 104 | -0.0875 | 0.0693 | 0.2069 |
| SCZ | Weighted median | 104 | -0.0925 | 0.1000 | 0.3552 |
| SCZ | Robust adjusted profile score (RAPS) | 104 | -0.0908 | 0.0725 | 0.2106 |
| BD | MR Egger | 11 | 1.9811 | 1.5069 | 0.2211 |
| BD | Inverse variance weighted | 11 | 0.1577 | 0.2436 | 0.5174 |
| BD | Weighted median | 11 | 0.1862 | 0.2707 | 0.4917 |
| BD | Robust adjusted profile score (RAPS) | 11 | 0.0991 | 0.2328 | 0.6703 |
| MDD | MR Egger | 55 | -2.4084 | 1.3146 | 0.0726 |
| MDD | Inverse variance weighted | 55 | 0.0468 | 0.2643 | 0.8594 |
| MDD | Weighted median | 55 | 0.3171 | 0.3765 | 0.3998 |
| MDD | Robust adjusted profile score (RAPS) | 55 | 0.0939 | 0.2811 | 0.7383 |
| AD | MR Egger | 31 | 0.1025 | 0.0997 | 0.3124 |
| AD | Inverse variance weighted | 31 | -0.0146 | 0.0510 | 0.7739 |
| AD | Weighted median | 31 | 0.0284 | 0.0794 | 0.7210 |
| AD | Robust adjusted profile score (RAPS) | 31 | -0.0269 | 0.0526 | 0.6093 |
| PD | MR Egger | 20 | 0.0110 | 0.2723 | 0.9683 |
| PD | Inverse variance weighted | 20 | -0.1185 | 0.0991 | 0.2319 |
| PD | Weighted median | 20 | -0.0948 | 0.1191 | 0.4258 |
| PD | Robust adjusted profile score (RAPS) | 20 | -0.1148 | 0.1009 | 0.2551 |

NSNP, number of instrumental SNPs selected; beta, effect of disorders on S100B; se, standard error for beta; Pval, p value for MR causal effects. Since only two SNPs met our criteria as instrumental SNPs for ASD, we did not perform analysis for this disorder.
